# A Pedagogical Reinforcement of the Ideal (Hard Sphere) Gas Using a Lattice Model: From Quantized Volume to Mechanical Equilibrium

**DOI:** 10.3390/e28010045

**Published:** 2025-12-30

**Authors:** Rodrigo de Miguel

**Affiliations:** Norwegian University of Science and Technology, 7034 Trondheim, Norway; rodrigo.demiguel@ntnu.no

**Keywords:** ideal gas, lattice models, volume quantization

## Abstract

Due to their simplicity and ease of visualization, lattice models can be useful to illustrate basic concepts in thermodynamics. The recipe to obtain classical thermodynamic expressions from lattice models is usually based on invoking the thermodynamic limit, and the ideal gas law can easily be obtained as the density of non-interacting particles vanishes. We present a lattice-based analysis that shows that, when a gas consisting of non-interacting particles evolves towards mechanical equilibrium with the environment, the ideal gas law can be obtained with no recourse to unnecessary assumptions regarding the size or particle density of the lattice. We also present a statistical mechanical analysis that considers a quantized volume and reproduces the process obtained for the discrete lattice model. We show how the alternative use of a well-known and accessible model (the non-interacting lattice gas) can give microscopic insights into thermal systems and the assumptions that underlie the laws used to describe them, including local vs. global equilibrium, irreversible processes, and the sometimes subtle difference between physical assumptions and mathematically convenient approximations.

## 1. Introduction

Two strategies stand out as most popular when introducing aspiring scientists to the fascinating realm of microscale physicochemical phenomena [1]: *thermodynamics first* and *quantum first*. A clear advantage of starting with quantum theory is that it provides students with a firm theoretical (and experimentally verified) submicroscopic foundation for the otherwise purely macroscopic theory of thermodynamics. While this should be a manageable challenge for the traditional physics student, physical science students in interdisciplinary programs may display a ‘negative disposition’ towards quantum theory, which they find ‘complicated, abstract, with a different logic’ and ‘with a lot of complicated mathematics’, in contrast to the apparently less daunting ideas of classical thermodynamics [2]. Indeed, despite the advantages of starting with quantum theory, it seems that many interdisciplinary textbooks favor the *thermodynamics first* approach [3].

An alternative approach to the introduction of thermodynamics advocated by many authors [4,5,6,7,8,9,10,11] is to bypass the submicroscopic considerations of quantum theory and develop classical thermodynamics in terms of the statistical behavior of particles. This provides the student with intuition in terms of familiar microscopic entities (atoms, molecules) as the basis of fundamental properties, like internal energy and entropy, around which classical thermodynamics is constructed. Indeed, the importance of properly grasping the concept of entropy has long been acknowledged by the larger teaching community in physics and chemistry, which has long engaged in discussions regarding its importance, frequent misconceptions, and various means of introducing it (see, e.g., refs. [12,13,14,15,16,17,18,19,20,21,22]).

When it comes to introducing entropy in terms of statistics, lattice models can be especially convenient. In refs. [23,24], Langbeheim et al. argued that the the lattice gas model has both a scientific and pedagogical advantage over the continuum model, and they described an interdisciplinary freshman course on soft matter where the ideas of thermodynamics are derived from the lattice gas. More recently, in ref. [25], Zhang presented a lattice model that can be used to introduce the concepts of entropy, free energy, and thermodynamic equilibrium based on basic probability. At a more advanced level, Dill and Bromberg showed how lattice models may be used as a basis to introduce many thermodynamic concepts at a level suitable for upper-division undergraduates across different study programs [26], and Åstrand and de Miguel have shown how lattice models and volume quantization may be used to merge the configurational and translational entropies [27,28].

The recipe to obtain classical thermodynamic expressions from lattice models is usually based on invoking the thermodynamic limit, and a result that is obtained with certain ease and elegance is the ideal gas law (see p. 98 in ref. [26]). In this work, we offer an approach that, although based on a lattice model, does not require unnecessary assumptions about the size or density of the lattice in order to reproduce the ideal gas law. Instead, we consider a process by which the system and the surroundings achieve mechanical equilibrium by exchanging ‘volume quanta’ and adjusting the the volume of the lattice sites. This reproduces ideal gas behavior without recourse to physical assumptions (such as infinite size and vanishing density), which should not be necessary when particles are already assumed to not interact, giving physical insight into the concept of *thermodynamic limit*.

The remainder of this paper is organized as follows. We introduce a simple discrete analysis of a lattice gas of non-interacting particles coming to mechanical equilibrium with its surroundings. We show that, once final equilibrium is achieved, the ideal gas law results with no recourse to assumptions about the size or particle density of the lattice. We then reconcile the discrete process with an accessible (yet somewhat more advanced) thermostatistical analysis that allows for volume quantization. We then provide some notes on the instructional context before closing with a discussion and some concluding remarks.

## 2. A Lattice Gas at Mechanical Equilibrium with Its Surroundings

We consider a gas at mechanical equilibrium with its surroundings (at pressure *p* and temperature *T*). If the internal energy and the number of particles are constant, from the thermodynamic identity dE=TdS−pdV+μdN, we conclude that

(1) $${\left(\frac{\partial S}{\partial V}\right)}_{E,N}=\frac{p}{T},$$

where *E*, *S*, *V* and *N* are, respectively, the energy, entropy, volume and number of particles in the gas. This is the general condition for mechanical equilibrium, and it is the analog of expressions ${\left(\partial S/\partial E\right)}_{V,N}=1/T$ and ${\left(\partial S/\partial N\right)}_{E,V}=-\mu /T$, which apply to thermal and diffusive equilibria, respectively (see, e.g., pp. 99–106 in ref. [26] or p. 120 in ref. [29]).

### 2.1. Sampling Local Equilibrium States: A Discrete Thermostatistical Analysis of a Non-Interacting Gas

In the following, we consider an adiabatic scenario where the system and the bath only exchange volume, not heat. This may be illustrated as a gas in a container (e.g., a cylinder) with movable boundaries (e.g., a piston) surrounded by an adiabatic jacket. Alternatively, one may assume that the system and the bath have already achieved thermal equilibrium (equal temperature), such that no further heat is exchanged. We choose to model the gas as a lattice gas consisting of *N* particles and m>N sites (all sites with equal volume), each of which can host one particle. We assume that the particles do not interact other than by volume exclusion, and each of the particles has an intrinsic volume *b*.

The number of configurational microstates is given by mN, and the resulting entropy is given by Boltzmann’s formula

(2) Sm=kBlnmN=kBlnm!N!(m−N)!,

where $k_{B}$ is the Boltzmann constant. Since there are *m* lattice sites, the volume of the gas is given by

(3) $$V_{m}=m\left(b+\nu_{0}\right),$$

where $\nu_{0}$ is the excess volume that each lattice site has beyond the minimum volume *b* needed to host one particle.

Given the quantization present in the model, we choose, for now, to write the equilibrium condition as a ratio of finite differences,

(4) $$\frac{\Delta V}{\Delta S}=\frac{V_{m}-V_{m-1}}{S_{m}-S_{m-1}}=\frac{T}{p},$$

or, invoking Equations (2) and (3),

(5) $$\underset{\mathrm{gas}}{\underset{︸}{\frac{b+\nu_{0}}{k_{B}\ln\left[\frac{m}{m-N}\right]}}}=\underset{\mathrm{reservoir}}{\underset{︸}{\frac{T}{p}}}.$$

The left side of Equation (5) represents the state (ΔV/ΔS) of the gas, and the right-hand side reflects the temperature-to-pressure ratio of the volume reservoir. This equality implies mechanical equilibrium between the gas and the environment, and any inequality would represent a departure from equilibrium.

When a system and a reservoir are at equilibrium, quanta may still flow between them in the form of fluctuations that cause momentary departures from equilibrium. It is these departures from equilibrium that allow systems to sample alternative states as they evolve from local to global equilibrium in their quest to maximize entropy subject to the environmental constraints imposed by the infinite reservoir. Note: This is the essence of Lars Onsager’s regression principle for irreversible processes in equilibrium systems [30,31,32,33].

If, in such a fluctuation, a quantum of volume were to flow from the system and out into the reservoir (i.e., m→m−1), the system’s volume (3) and entropy (2) would decrease, increasing the system’s pressure and causing a departure from equilibrium:

(6) $$\frac{b+\nu_{0}}{k_{B}\ln\left[\frac{m-1}{m-1-N}\right]}<\frac{T}{p}.$$

When this happens, equilibrium is easily restored, and the lost entropy regained, by simply having the volume quantum flow back into the system.

In the following, we focus on the opposite scenario, i.e., departures from equilibrium caused by volume quanta flowing *into* the system. When the system receives an additional quantum of volume from the reservoir, its pressure decreases, resulting in a slight departure from equilibrium:

(7) $$\frac{b+\nu_{0}}{k_{B}\ln\left[\frac{m+1}{m+1-N}\right]}>\frac{T}{p}.$$

Yet, as opposed to the fluctuation described above, in this process, the system has gained entropy. The system may then restore equilibrium without losing its newly gained entropy by adjusting the size of its volume quanta (i.e., the volume of the lattice sites) from $\left(b+\nu_{0}\right)$ to a smaller $\left(b+\nu_{1}\right)$, such that

(8) $$\frac{b+\nu_{1}}{k_{B}\ln\left[\frac{m+1}{m+1-N}\right]}=\frac{T}{p}.$$

If a new quantum of volume, now with size $\left(b+\nu_{1}\right)$, is received from the external volume reservoir (i.e., m+1→m+2), a new departure from equilibrium results:

(9) $$\frac{b+\nu_{1}}{k_{B}\ln\left[\frac{m+2}{m+2-N}\right]}>\frac{T}{p}.$$

Then, as before, the volume sites in the system may become smaller $\left(\nu_{2}<\nu_{1}\right)$ so that equilibrium is again recovered without losing entropy:

(10) $$\frac{b+\nu_{2}}{k_{B}\ln\left[\frac{m+2}{m+2-N}\right]}=\frac{T}{p}.$$

**Figure 1:**
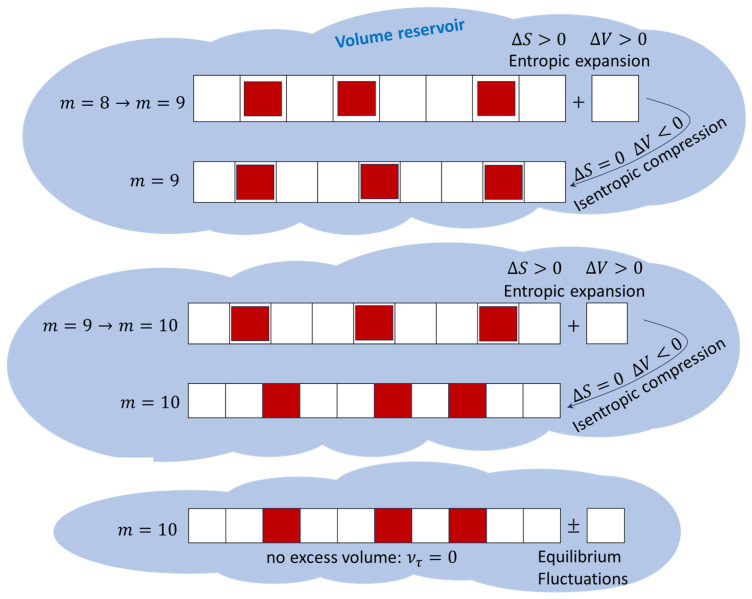
The system is modeled as a lattice consisting of volume sites (white squares), each of which can host one particle (solid red square). The system absorbs a quantum of volume (a new lattice site); this increases the configurational entropy and decreases the system’s pressure. Thereafter, the volume of the lattice is isentropically reduced (by reducing the volume of each lattice site), increasing the system’s pressure. The process is repeated until the lattice sites (white squares) cannot become any smaller.

This process may only be repeated τ times,

(11) $$\frac{b+\nu_{\tau }}{k_{B}\ln\left[\frac{m+\tau }{m+\tau -N}\right]}=\frac{T}{p},$$

until the site volume, given by

(12) $$b+\nu_{\tau }=\frac{k_{B}T}{p}\ln\left[\frac{m+\tau }{m+\tau -N}\right],$$

cannot become any smaller (see Figure 1), i.e., until the excess volume $\nu_{\tau }=0$, which implies that the final volume (3) of the system is given by

(13) $$V=V_{m+\tau }=\left(m+\tau \right)\left(b+\nu_{\tau }\right)=\left(m+\tau \right)b,$$

and the number of lattice sites (setting $\nu_{\tau }=0$ in Equation (12)) is given by

(14) $$m+\tau =\frac{N}{1-e^{-bp/k_{B}T}}.$$

Combining the last two expressions, we obtain the equation of state

(15) $$V=\frac{bN}{1-e^{-bp/k_{B}T}},$$

which may be unfolded as

(16) $$\frac{V}{N}=\frac{k_{B}T}{p}+\frac{1}{2}b+O\left(b^{2}\right).$$

Expression (16) only differs from the ideal gas of point particles due to the volume factor *b*. This is an important physical factor to acknowledge as only if this factor is non-zero (positive) will the particles ever exchange energy and the gas ever reach equilibrium; otherwise, all particles would keep their initial velocities forever. Indeed, interactions (even minimal ones) are necessary for the emergence of thermodynamic behavior in gases. Only after this is noted may we take further approximations to fit the real system of interest. Indeed, to first order in *b*, we obtain a Van der Waals gas without attractive interactions (a gas of hard spheres). And, for negligibly small *b*, we obtain the more familiar ideal gas model.

Regardless of the level of idealization, gas laws are equilibrium models, and, as such, they describe the system at the end of an equilibration process that maximizes the entropy according to some constraints. Other than non-interacting particles, the only physical assumption invoked above is a sufficiently long time for the system to fully equilibrate with the environment.

### 2.2. Discrete Volume, Entropy, and Irreversibility

We have described above how the system undergoes a series of steps as it evolves towards final equilibrium. Each step in the process is completely adiabatic and consists of two stages (see Figure 1):

- An irreversible entropic expansion via the absorption of a quantum of volume (a new lattice site);
- An isentropic compression whereby the volume of each lattice site is reduced.

The volume change in this two-stage process is obtained from Equation (3),

(17) $$V_{m+t}-V_{m+t-1}=\left(m+t\right)\left(b+\nu_{t}\right)-\left(m+t-1\right)\left(b+\nu_{t-1}\right),$$

and, invoking Equation (12), it becomes

(18) $$V_{m+t}-V_{m+t-1}=\frac{k_{B}T}{p}\ln\left[{\left(\frac{m+t}{m+t-N}\right)}^{m+t}\right]-\frac{k_{B}T}{p}\ln\left[{\left(\frac{m+t-1}{m+t-1-N}\right)}^{m+t-1}\right].$$

Rearranging the logarithmic terms, and then invoking Equations (2) and (12), this expression may be written as

(19) $$V_{m+t}-V_{m+t-1}=\frac{T}{p}\left(S_{m+t}-S_{m+t-1}\right)+\left(m+t\right)\left(\nu_{t}-\nu_{t-1}\right).$$

The first term on the right-hand side of (19) represents the first point noted above, i.e., the arrival of a new amount of volume that increases the system’s configurational entropy given by Equation (2). The second term represents the second point, i.e., an isentropic adjustment of the volume quanta present in the system: the lattice sites change from size $\left(b+\nu_{t-1}\right)$ to size $\left(b+\nu_{t}\right)$. As times goes on, the isentropic adjustments of the volume quanta become increasingly insignificant, and the last term in (19) vanishes, resulting in the state of maximum entropy, which is compatible with the mechanical equilibrium condition ΔV/ΔS=T/p given by Equation (4).

## 3. A More Advanced Thermostatistical Analysis Allowing for Discrete Volume

In the following, we show how a continuous version of Equation (19) may indeed be obtained by performing a classical statistical mechanical analysis if it considers discrete volume in addition to discrete energy and particle numbers in the augmented Boltzmann factor. This supplementary analysis is more advanced, as it invokes tools from statistical mechanics.

The average value *X* of an extensive variable whose intensive conjugate is η is generally given by (see, e.g., pp. 69–70 in ref. [34])

(20) $$X=-k_{B}\frac{\partial \ln\Xi }{\partial \eta },$$

where Ξ is the generalized partition function. Note: Typical {X;η} conjugate pairs are {E;1/T}, {V;p/T} and {N;−μ/T}. However, depending on the system, other pairs may exist. For example, in systems where the possible exchange of electric charge is considered, there will be {Q;ϕ/T}, where Q is the charge and ϕ is the equilibrium electric potential.

Following this expression, the average volume *V* of the system is given by

(21) $$V=-k_{B}\frac{\partial \ln\Xi }{\partial \gamma^{-1}},$$

where $\gamma^{-1}\equiv p/T$ is the intensive conjugate to the volume, and

(22) $$\Xi \equiv \sum_{i}e^{-E_{i}/k_{B}T}\;e^{-pV_{i}/k_{B}T}\;e^{\mu N_{i}/k_{B}T}$$

is a generalized partition function allowing not only for energy quantization but also for a discrete volume and number of particles.

We now consider a scenario where no heat is exchanged (i.e., no transitions between energy levels) and neither are particles. The only extensive quantity exchanged is volume. The system (a lattice gas) exchanges volume quanta (lattice sites) with the environment to reach mechanical equilibrium. Since we are assuming that the system is closed to heat and particle exchange, then $E_{i}$ and $N_{i}$ are constants independent of the states *i* available to the system, and the partition function (22) may be written as

(23) $$\Xi =c\cdot Z_{v},$$

where *c* is some constant and

(24) $$Z_{v}\equiv \sum_{i}e^{-V_{i}/k_{B}\gamma }.$$

Since the gap between consecutive ‘volume levels’ is dependent on γ (see Equation (12)), the values of the levels $V_{i}$ themselves depend on γ. Then, taking the derivative, the volume (21) becomes

(25) $$V=\frac{1}{Z_{v}}\sum_{i}\left(V_{i}-\gamma \frac{\partial V_{i}}{\partial \gamma }\right)e^{-V_{i}/k_{B}\gamma }.$$

Expression (25) differs from the expected expression only by the presence of the second term in the parentheses. While the value of $V_{i}$ (present in the factor $e^{-V_{i}/k_{B}\gamma }$) determines the probability that the system adopts a given state (the volume state composed of *i* lattice sites), the whole $V_{i}-\gamma \partial V_{i}/\partial \gamma$ determines the actual contribution made to the system’s volume by that state once it is adopted. The reader should note that this nuance (i.e., considering γ-*dependent volume levels*) is not unique to the volume; indeed, an expression similar to Equation (25) for the average internal energy (where *V* becomes *E* and γ becomes *T*) was first justified and introduced by Elcock and Landsberg in ref. [35] (see Equation 2.13 therein), and it is briefly mentioned in Pathria and Beale’s landmark textbook on statistical mechanics (see ch. 3, footnote 1, in ref. [36]).

(As a side note, the reader should know that, far from being an eccentricity, the just mentioned framework of *temperature-dependent energy levels* [35] was directly invoked in early studies of semiconductors [37] and bosonic systems [38,39]. Indeed, temperature-dependent energy levels have since become ubiquitous in the study of these systems (see, e.g., refs. [40,41] for semiconductors and ref. [42] for bosonic systems). The theory has also been applied to superconductivity [43], optomechanical oscillators [44], thermoelectric phenomena [45,46], small-system thermalization [47], heat engines [48] and even information theory [49]. More recently, Landsberg’s idea of a statistical mechanics with *temperature-dependent* energy levels has reappeared in a framework known as *statistical mechanics at strong coupling* [50,51] and been applied to describe nanomechanical systems [52], nanoscale interfacial phenomena [53], thermophilic motion [54] and perturbation theory [55]).

At present, we focus exclusively on volume exchange at constant *E*. For simplicity, we write Equation (25) as

(26) $$V=\langle V_{i}\rangle -\gamma \langle \frac{\partial V_{i}}{\partial \gamma }\rangle ,$$

where $\langle \cdot \rangle$ represents the weighted average over all volume states. The second term on the right-hand side of Equation (26) is a correction resulting from the dependence of the volume levels $V_{i}$ on γ. In line with Onsager’s local linearity principle [33], we may, to first order, assume that this perturbation is linear in γ such that $\langle \partial V_{i}/\partial \gamma \rangle$ is constant.

Taking increments on both sides yields

(27) $$d\langle V_{i}\rangle =dV+\langle \frac{\partial V_{i}}{\partial \gamma }\rangle d\gamma ,$$

which, invoking (1), becomes

(28) $$d\langle V_{i}\rangle =\gamma dS+\langle \frac{\partial V_{i}}{\partial \gamma }\rangle d\gamma .$$

Note that expression (28) is the continuous analog to the discrete expression (19), and it describes the following process. When a system comes into mechanical contact with a reservoir, volume is absorbed. The arrival of volume increases the system’s entropy by an amount dS. Thereafter (second term on right-hand side), the system reduces the size of the gaps in the volume spectrum $V_{i}$ in order to reduce its volume and recover its pressure. The volume is reduced isentropically, and the gap $d\langle V_{i}\rangle$ between the initial and final volume is no longer given by the original γdS but instead by a smaller $\gamma dS+\langle \partial V_{i}/\partial \gamma \rangle d\gamma$. In the thermodynamic (large system) limit, the system’s spectrum $V_{i}$ becomes independent of γ (i.e., of the externally imposed T/p), and the last term vanishes as a result.

(Note: If multiplied by *p*, expression (28) is the work analogue of the modified heat law $dQ=TdS+\langle \partial E_{i}/\partial T\rangle dT$ first proposed by Shental and Kanter in ref. [49] (see Equation (3) therein) and more recently invoked in, e.g., refs. [45,46,47,48,54].)

## 4. Instructional Context

The process presented in Section 2 is not meant as a substitute for traditional introductions to ideal gases. Instead, it offers a framework within which to revisit the familiar ideal gas law, which students have presumably seen earlier using kinetic theory (see, e.g., §18.3 in the introductory physics text ref. [56]) and classical thermodynamics (see, e.g., §2.2 in the physical chemistry text ref. [57]).

The process in Section 2 is not only mathematically accessible to the typical upper-division physical science student. It is also very suitable for computational exploration using simple software. One may set environmental parameters (temperature *T* and pressure *p*, or, more simply, their ratio T/p) and system parameters (number of particles *N* and intrinsic particle volume *b*). Then, one models the system as a lattice gas by assigning an initial number of lattice sites m>N with an excess volume $\nu_{0}>0$ at each site. Then, let the equilibration process begin in discrete time steps that increase the number of lattice sites (i.e., the configurational entropy) and simultaneously decrease the site volume (to reduce the total volume and stay at equilibrium) until the excess volume is zero (i.e., entropy may no longer be increased without irremediably departing from equilibrium). This allows for an exploration of which physical conditions (T/p) cause the second term on the right-hand side of (19) to become negligible most rapidly, giving insight into non-equilibrium processes and their relations to equilibrium expressions such as ΔV/ΔS=T/p.

In contrast to Section 2, the framework presented in Section 3 is not discrete, and it is admittedly more advanced, as it assumes some familiarity with the tools of statistical mechanics. It complements the discrete analysis, and it is appropriate for students familiar with concepts like ensemble averages and the partition function, i.e., more advanced students who have explored the ideal gas using traditional statistical mechanics (see, e.g., §5.3 in the statistical mechanics text ref. [58]). Yet, again, it presents a new opportunity to reflect over the physical conditions that cause the second term on the right-hand side of (28) to vanish, i.e., the thermodynamic limit, where the system is so large that its volume (or energy) levels are only determined by its own internal structure and not at all perturbed by external factors like γ (or *T*).

(Note: The parentheses in the last sentence are a reference to the framework of temperature-dependent energy levels mentioned above. It has been shown that the energy landscapes of small systems (i.e., systems not properly described by the thermodynamic limit) are perturbed by the temperature of the environment that they are at equilibrium with and that, for large systems, this perturbation is negligible (i.e., the system’s temperature is fully determined by its own intrinsic energy landscape), rendering negligible the last term of an expression analogous to (28), where *V* becomes *E* and γ becomes *T*. See, e.g., refs. [50,55].)

## 5. Discussion and Conclusions

Lattice models have a wide range of applications in chemical physics, as they provide simplified, discrete representations of space in which interactions, motion, and reactions can be studied with controlled assumptions that balance microscopic realism and analytical (or computational) tractability, simplifying the description of collective phenomena arising from simple local interactions. Lattice models allow us to isolate essential degrees of freedom while retaining geometry and dimensionality. For this reasons, they are widely used across active areas like magnetism [59,60], binary mixtures [61], adsorption–desorption equilibria [62], nucleation [63,64], interfacial phenomena [65], polymers [66], gels [67] and biomolecules [68,69,70].

Beyond the descriptive power of lattice models to approach demanding problems at the edges of science, these models also offer pedagogical insight in earlier learning stages. The authors of refs. [23,24] argue for the compelling pedagogical advantages of the lattice model over the continuum model of gases. In contrast to the continuum model, volume exclusion is a natural feature of the lattice model, preventing different particles from being at the same place at the same time. Moreover, the visual characteristics of the lattice model support the abstract mathematical derivations that it motivates. Ref. [26] takes this concept to the next level and constitutes a coherent upper-division textbook in statistical thermodynamics built largely upon lattice models.

Models are but symbolic representations of physical behavior. Yet, in the making of models, one may become confused by the sometimes subtle difference between simplifications motivated by mathematical convenience (such as the continuity approximation) and physical assumptions (such as low density). When modeling the behavior of non-interacting particles as a lattice gas, the usual procedure is to start with two mathematical approximations: first, the entropy and the volume are taken to be continuous functions of the number of lattice sites so that derivatives can be taken; second, the number of lattice sites and the number of particles are assumed to be large so that Stirling’s approximation can be applied to the entropy (these mathematical approximations are reminiscent of a large-system assumption, and, while this may be physically reasonable in many contexts, it is not necessary to model a gas of non-interacting particles). Finally, a more explicitly physical low-density assumption is made, and the ideal gas law results (see p. 98 in ref. [26]). While the derivation is elegant and the approximations physically meaningful, they are not necessary in light of the most fundamental premise in the chosen model, i.e., non-interacting particles.

The supplementary method by which to obtain the ideal gas law that we share in this work illustrates more clearly the benchmark for the physical conditions under which real gases may be treated as ideal, and it leads naturally to the conclusion that *the reason why the real gas must have low density is that attractive interactions have to vanish, which is the only physical premise behind the ideal expression (16).*

This derivation also illustrates that the general validity of thermodynamic expressions is not necessarily a large-system effect, but rather an effect where size plays a role only as it relates to interactions. Indeed, in the absence of interactions, size is not important. Furthermore, whether or not classical expressions apply is not solely dependent on the sheer size of a system but rather on how the system’s size relates to its interactions [53]. This explains why classical thermodynamic expressions sometimes apply at the nanoscale (see, e.g., refs. [71,72,73]), yet they are inadequate for very large systems if these are subject to long-range interactions [74].

Another takeaway from this framework is that it offers microscopic intuition regarding the often challenging idea of ‘local equilibrium’ in non-equilibrium (i.e., irreversible) processes. Indeed, the equilibrium ideal gas law is arrived at by discretizing matter and volume and then allowing the system to evolve towards final mechanical equilibrium in a series of steps, each at local equilibrium. This offers a simple yet insightful illustration of how an irreversible process may be analyzed as a grand sequence of steps at local equilibrium [33].

In conclusion, in this work, we offer a pedagogical reinforcement of the familiar ideal gas law and the physical context it describes. The framework that we propose is based on a mathematically accessible discrete model (the lattice gas), and the physical assumptions on the system are now limited to (i) non-interacting particles and (ii) mechanical equilibrium with the environment. This illustrates that asymptotic approximations are only important in the presence of interactions, and it gives insight into local vs. final equilibrium in irreversible processes, all within the framework of a familiar (and ever pedagogically insightful) model like the ideal gas.

## Data Availability

The original contributions presented in this study are included in the article. Further inquiries can be directed to the corresponding author.
